# Psychosocial job strain and polypharmacy: a national cohort study

**DOI:** 10.5271/sjweh.3914

**Published:** 2020-10-30

**Authors:** Edwin CK Tan, Kuan-Yu Pan, Linda L Magnusson Hanson, Johan Fastbom, Hugo Westerlund, Hui-Xin Wang

**Affiliations:** 1 The University of Sydney, Faculty of Medicine and Health, School of Pharmacy, Sydney, New South Wales, Australia; 2 Aging Research Center, Department of Neurobiology, Care Sciences and Society, Karolinska Institutet and Stockholm University, Stockholm, Sweden; 3 Stress Research Institute, Department of Psychology, Stockholm University, Stockholm, Sweden; 4 Centre for Medicine Use and Safety, Faculty of Pharmacy and Pharmaceutical Sciences, Monash University, Parkville, Australia; 5 Amsterdam University Medical Center, Vrije Universiteit, Department of Psychiatry, Amsterdam Public Health Research Institute, The Netherlands

**Keywords:** coping, epidemiology, job control, job demand, occupational stress, stress

## Abstract

**Objectives:**

Psychosocial job strain has been associated with a range of adverse health outcomes. The aim of this study was to examine the association between psychosocial job strain and prospective risk of polypharmacy (the prescription of ≥5 medications) and to evaluate whether coping strategies can modify this risk.

**Methods:**

Cohort study of 9703 working adults [mean age 47.5 (SD 10.8) years; 54% female] who participated in the Swedish Longitudinal Occupational Survey of Health (SLOSH) at baseline in 2006 or 2008. Psychosocial job strain was represented by job demands and control, and measured by the Swedish version of the demand–control questionnaire. The outcome was incidence of polypharmacy over an eight-year follow-up period. Information on dispensed drugs were extracted from the Swedish Prescribed Drug Register. Logistic regression was used to estimate the association of job strain status with polypharmacy, adjusted for a range of confounders.

**Results:**

During the follow-up, 1409 people developed polypharmacy (incident rate: 20.6/1000 person-years). In comparison to workers with low-strain jobs (high control/low demands), those with high-strain jobs (low control/high demands) had a significantly higher risk of incident polypharmacy (OR 1.40, 95% CI 1.04–1.89). The impact of high-strain jobs on developing polypharmacy remained among those with covert coping strategies (ie, directed inwards or towards others) but not among those with open coping strategies (ie, primarily directed toward the stressor).

**Conclusions:**

Workers in high-strain jobs may be at an increased risk of polypharmacy. Open coping strategies may reduce the negative impact of psychosocial job strain on risk of polypharmacy.

Polypharmacy, the use of multiple medications by one individual, is increasing worldwide among those with multimorbidity (1). Polypharmacy has been highlighted as a key focus area by the World Health Organization in their global strategy to optimize medication use without harm (2). While there is no standard definition for polypharmacy, the most common is the concurrent use of ≥5 medications by a single individual (3). Although prescription drug use is highest among older people, the prevalence of polypharmacy has increased across all age groups over the last decade. In the US, the prevalence of polypharmacy increased from an estimated 8.2% in 1999–2000 to 15% in 2011–2012. Among those 20–39 years of age, the prevalence was 3.1% and 15% among 40–64-year olds (4). In 2015–2016, 60% of US adults aged 40–59 used ≥1 prescription drugs, and 15% used ≥5 (5). In Sweden, the prevalence of polypharmacy increased from 17% in 2006 to 19% in 2014 in the general population. The rate of polypharmacy was 8.5% among those <60 years (6). With growing medication use among those of working age, the contribution of working life to polypharmacy warrants further investigation.

Polypharmacy is associated with a range of adverse outcomes including inappropriate medication use, poor adherence, medication errors and drug interactions (7). Polypharmacy has also been linked to increased healthcare costs, and increased risks for hospitalization and mortality (1, 8). While a range of sociodemographic and socioeconomic risk factors for polypharmacy have been investigated (9), there is a paucity of research on the role of the psychosocial work environment (10). To date, no studies have investigated the association between psychosocial work stress and future risk of polypharmacy.

The psychosocial work-environment has been widely studied in terms of job strain – a situation with high psychological demands or pressures combined with low control in meeting those demands – and is assumed to be a risk factor for physical and mental health (11). Job strain has been reported to be associated with a range of adverse health outcomes including depression (12), cardiovascular disease (13, 14), diabetes (15), hypertension (16, 17), sleep disturbance (18), and musculoskeletal disorders (19, 20). Coping, defined as ongoing cognitive and behavioral efforts to manage psychological stress (21), may be employed to reduce the negative consequences of work stress. Coping may be broadly categorized into problem-focused (active efforts to resolve a problem) or emotion-focused (aims to reduce emotional discomfort) (22) and coping strategies may be primarily directed towards the stressor (open coping) or directed inwards or towards others (eg, family or friends) (covert coping) (23, 24). Coping has been previously found to have a moderating effect in the association between psychosocial work stress and various health and psychological outcomes (25–27). While certain strategies may be more beneficial than others, there remains a lack of clarity regarding coping’s moderating role between job strain and a broader range of health outcomes.

The aim of this study was to examine the association between psychosocial job strain and prospective risk of polypharmacy (the use of ≥5 medications) and to evaluate whether coping strategies can modify this risk.

## Methods

### Study population

This was a data linkage study based on participants in the Swedish Longitudinal Occupational Survey of Health (SLOSH), an ongoing study of an approximately nationally representative sample of the Swedish working population (www.slosh.se) (28). Since 2006, a mailed self-completion questionnaire with two versions has been distributed biennially. Recipients who were in paid work ≥30% of full-time answered one version and recipients who temporarily or permanently worked <30% of full-time answered the other version. Statistics Sweden carried out the data collection, and a submitted response to the survey confirmed informed consent. The questionnaire asked extensive information on occupation, psychosocial work environment, work organization, health, and health-related complaints. The overall response rate to the questionnaires was 65% in 2006 (5985 respondents; 5141 to the ‘worked ≥30% of full-time’ and 844 to the ‘worked <30% of full-time’ questionnaire) and 61% in 2008 (11 441 respondents; 9756 to the ‘worked ≥30% of full-time’ and 1685 to the ‘worked <30% of full-time’ questionnaire) (28).

This study included participants who worked ≥30% of full-time when entering SLOSH in the year 2006 (N=5141) or 2008 (N=5170). Thus, there were a total of 10 311 individuals in gainful employment at baseline [mean age 47.8 (SD 10.8) years; 54% female]. We defined baseline as the first six months in 2006 and 2008 for the two cohorts, respectively. All the dates of study entry fell within this range. Prevalent cases were those who had polypharmacy during these six months. After excluding prevalent polypharmacy cases (≥5 drugs, N=197) and people with missing occupational information (N=411), 9703 participants were included in the analytical sample.

### Exposure

Psychosocial job strain was represented by job demands and control, and measured by the Swedish version of the demand–control questionnaire (DCQ) in SLOSH. The DCQ is widely used and has satisfactory psychometric properties (29, 30). Psychosocial demands at work were measured with five questions: (i) do you have to work very fast? (ii) Do you have to work very intensively? (iii) Does your work demand too much effort? (iv) Do you have enough time to do everything? (v) Does your work often involve conflicting demands? Job control was represented by decision authority and skill discretion and was assessed by six items from the DCQ: (i) do you have a choice in deciding how to do your work? (ii) Do you have a choice in deciding what you do at work? (iii) Do you have the possibility of learning new things through your work? (iv) Does your work demand a high level of skill or expertise? (v) Does your work require ingenuity? (vi) Do you have to do the same thing over and over again? These items were quantified on a scale from 1=“yes, often” to 4=“no, hardly ever/never”. Except for answers to question 4 of the demands dimension and question 6 of the control dimension, the responses to the other nine questions were converted into 1=“no, hardly ever/never” to 4=“yes”. Response scores for job demands and job control were averaged, respectively. Thus, the response scores were presented in an ascending fashion (ie, higher scores referred to higher control/demands). Following Karasek’s job strain model (11), we combined job control and demands into four categories of job strain: low-strain jobs (low demands, high control); passive jobs (low demands, low control); active jobs (high demands, high control); and high-strain jobs (high demands, low control). Psychosocial job strain was assessed at baseline only as it has been previously shown that levels of job demands and control remain relatively unchanged over time in the SLOSH cohort (31).

### Outcome

The main outcome was the incidence of polypharmacy over an eight-year follow-up period. Information on dispensed drugs were extracted from the Swedish Prescribed Drug Register. This register contains data with unique patient identifiers for all prescriptions dispensed by pharmacies to the whole population of Sweden. Information on medication included the exact date of drug purchasing, drug name, dosage and quantity. All drugs were classified according to the anatomical therapeutic chemical (ATC) classification system (32). Polypharmacy was defined as the prescription of ≥5 medications and “excessive polypharmacy” was defined as the prescription of ≥10 medications. Data on drugs dispensed every quarter from the date of study entry were extracted and used to calculate quarterly polypharmacy outcomes each year over an eight-year period. The assessment of polypharmacy was based on the current drug use the last day of each quarter, by using a point prevalence method described previously (33).

### Covariates

Characteristics were assessed by age and gender. Socioeconomic variables included the highest level of education (categorized into elementary, high school and university) and occupational class (blue- or white-collar worker). Age, gender and education were derived from national administrative registers and occupational class from the survey question: “To which occupational category do you belong?” with responses: (i) worker, (ii) clerical, (iii) line manager, and (iv) other. The first response was categorized as blue-collar worker and the remaining as white-collar worker. The presence of chronic diseases was derived from linked data from the National Patient Register which contains information for all inpatient and specialized outpatient visits in Sweden. All chronic diseases were defined according to International Classification of Diseases, 10^th^ revision (ICD-10), and grouped into broad categories of chronic conditions, as previously reported (34).

The questions on coping were based on a questionnaire originally developed for a US study on high blood pressure but later adapted for the Swedish working population, which has been tested extensively (24, 35). Information about coping strategies in 2006 was collected by the question: “During the last two years when you felt steamrolled or unfairly treated by your manager/managers or workmates, how have you reacted?” In 2008, this was changed to two separate questions: “How do you usually react if you are unfairly treated or get into conflict with a supervisor/manager?” and “How do you usually react if you are unfairly treated or get into conflict with a workmate?” In response to these questions there were four possible options that were graded on a scale, with 1=always to 4=never: (i) immediately made clear and clearly shown my feelings, (ii) suggested a compromise or other solution, (iii) kept quiet and brooded over it, and (iv) taken it out on my family/those closest to me. The first two responses represented open coping strategies, whilst the last two represented covert coping strategies. A sum score of coping strategies was calculated, ranging from 8–32, with higher scores representing more open coping strategies (24). Responses for the separate questions in 2008 were averaged. We further dichotomized scores using the median score for men and women separately.

### Statistical analysis

Baseline characteristics across job strain categories and between people with and without polypharmacy were compared using Chi-square (χ^2^) or one-way ANOVA followed by pairwise comparison with Bonferroni correction. Logistic regression was used to estimate the odds ratios (OR) and 95% confidence intervals (CI) for the association of job demands, job control and job strain status with polypharmacy. Logistic regression analyses were deemed more appropriate than Cox regression, as exact follow-up time to polypharmacy occurrence could not be determined (based on quarterly time points) and Cox proportionality assumptions did not hold across analyses.

Job control and job demands were treated as continuous variables first to examine linear associations with the outcome. To explore nonlinear associations, we created indicator variables for job control and job demands, respectively, using quartile distribution in men and women, separately. This was done to take into consideration gender differences in reporting perceived stress. The results from the quartile analyses revealed that the job controls and demands had different thresholds for polypharmacy, and the thresholds were similar between men and women. Thus we dichotomized job control (1^st^ quartile versus 2^nd^–4^th^ quartile) and demands (1^st^ and 2^nd^ quartile versus 3^rd^ and 4^th^ quartile), respectively, and created the four job strain categories, with low strain as the reference group.

Statistical interactions between job strain status and coping strategy, as well as potential effect modifiers such as sex, age, education, lifestyle factors, health status, and social support at work, were tested by introducing interaction terms in the model. In the case where a statistically significant interaction was detected, stratified analysis was further conducted to compare polypharmacy risks across strata.

All analyses were first adjusted for age, sex and follow-up time, and additionally controlled for potential confounders. Variables that were significantly associated with polypharmacy and job strain, including occupational class, education and number of chronic diseases, were kept in the model in order to obtain the independent effect of job strain on polypharmacy. Collinearity tests showed that the variance inflation factor (VIF) for all the covariates was <1.26, with a mean value of 1.15, indicating the fully adjusted model should not have collinearity problems and the regression estimates from such a model should be stable. Sleep quality, depressive symptoms, and lifestyle factors were not significantly associated with polypharmacy or job strain and thus excluded from the analysis. A description of these covariates is provided in the supplementary material (https://www.sjweh.fi/show_abstract.php?abstract_id=3914).

Several sensitivity analyses were carried out. First, we excluded people taking any drugs at baseline to include participants who were relatively healthier. Second, we redefined the outcome as multiple/repeated polypharmacy over the follow-up period. This was done in order to lower the risk of false positive polypharmacy and also evaluate chronicity of polypharmacy. Third, we repeated the analysis by excluding 185 individuals who were <26 years because the occurrence of polypharmacy was uncommon (<2.17%). We also performed stratified analysis by age <55 versus ≥55 years because the younger participants had a lower rate of polypharmacy (≤15.58%) than the older participants (≥21.68%). Fourth, we performed stratified analysis by occupation because both men and women work to a very large extent in Sweden, the labor market is quite gender segregated horizontally (men and women tend to have different occupations). Therefore, gender differences in demands and control could be due to actual differences between the jobs that men and women tend to do rather than the differences in reporting style as mentioned before. Finally, we used active job as a reference group as this has been reported in the occupational health literature and in studies of other health outcomes.

All reported P-values were two-sided, and P<0.05 was considered statistically significant. Data were computed using Stata SE 14.0 (StataCorp LP, College Station, TX, USA).

### Ethical considerations

This study was conducted with the approval of the Regional Research Ethics Board in Stockholm. Data were coded and anonymized before statistical analyses.

## Results

A total of 9703 participants were followed up to 31 December 2014 [mean follow-up time 7.1 (SD) 2 years]. Baseline characteristics of the study population by job strain status are summarized in table 1.

**Table 1:** Baseline characteristics of the study population by job strain status (N=9703). Missing data: education=48, occupational class=226, self-rated health=69, coping strategy=1261. [SD=standard deviation.]

Characteristics	Low Strain N=3440		Active N=3211		Passive N=1732		High Strain N=1320		P-value
			
	Mean (SD)	N (%)	Mean (SD)	N (%)	Mean (SD)	N (%)	Mean (SD)	N (%)	
Age (years)	48.2 (10.7)		47.6 (10.4)		46.6 (11.4)		46.9 (10.8)		<0.001
Female sex		1739 (50.6)		1729 (53.9)		982 (56.7)		790 (59.9)	<0.001
Education									
Elementary		545 (16.0)		308 (9.7)		392 (22.6)		306 (23.3)	
High school		1457 (42.6)		1170 (36.6)		1014 (58.6)		698 (53.2)	<0.001
University		1416 (41.4)		1716 (53.7)		325 (18.8)		308 (23.5)	
Occupational class									
Blue-collar worker		1243 (36.9)		827 (26.6)		1028 (60.3)		774 (59.7)	<0.001
White-collar worker		2125 (63.1)		2280 (73.4)		678 (39.7)		522 (40.3)	
Chronic diseases	0.6 (1.1)		0.6 (1.1)		0.6 (1.1)		0.7 (1.1)		0.07
Coping strategy									
Covert		1456 (49.6)		1663 (59.2)		937 (62.3)		790 (66.3)	<0.001
Open		1480 (50.4)		1146 (40.8)		568 (37.7)		402 (33.7)	

During the follow-up, 1409 (14.5%) people developed polypharmacy (prescription of ≥5 medications, incident rate: 20.6/1000 person-years). Incidence of polypharmacy varied across age groups: 19–39 years (N=113, 4.64%), 40–49 years (N=266, 9.79%), 50–54 years (N=230, 15.58%), 55–59 years (N=343, 21.6%), and 60–68 years (N=457, 30.5%). Of those with incident polypharmacy, 103 individuals developed excessive polypharmacy (prescription of ≥10 medications). As the incidence of excessive polypharmacy was low, we decided to run analyses focusing on polypharmacy. Baseline characteristics of the study population by polypharmacy outcome status are summarized in table 2. Those participants who developed polypharmacy were older and less educated, had greater number of chronic diseases and were less likely to use covert coping strategies.

**Table 2:** Baseline characteristics of the study population by incident polypharmacy status (N=9703). Missing data: education=48, occupational class=226, self-rated health=69, coping strategy=1261. [SD=standard deviation.]

Characteristics	Non-cases N=8294		Cases N=1409		P-value
	
	Mean (SD)	N (%)	Mean (SD)	N (%)	
Age (years)	46.5 (10.7)		53.8 (8.9)		<0.001
Female sex		4483 (54.1)		757 (53.7)	0.82
Education					
Elementary		1189 (14.4)		362 (25.7)	
High school		3759 (45.6)		580 (41.3)	<0.001
University		3302 (40.0)		463 (33.0)	
Occupational class					
Blue-collar worker		3298 (40.7)		574 (41.8)	0.44
White-collar worker		4806 (59.3)		799 (58.2)	
Chronic diseases	0.5 (0.9)		1.4 (1.7)		<0.001
Coping strategy					
Covert		4208 (58.0)		638 (53.9)	<0.01
Open		3051 (42.0)		545 (46.1)	

The most prevalent medications contributing to polypharmacy at follow-up were beta blockers (ATC code: C07A), lipid modifying agents (C10A), antithrombotic agents (B01A), antidepressants (N06A), hypnotics and sedatives (N05C), drugs for peptic ulcer and gastro-oesophageal disease (A02B), opioids (N02A), calcium channel blockers (C08C), angiotensin-converting enzyme (ACE) inhibitors (C09A), and other analgesics (N02B).

Compared with low job control, workers with high job control had a lower risk for polypharmacy (OR 0.74, 95% CI 0.59–0.92); however, there was no association between job demands and polypharmacy (table 3). In comparison to workers in active jobs, those in high-strain jobs had a significantly higher risk of incident polypharmacy (OR 1.40, 95% CI 1.04–1.89) (table 4).

**Table 3:** Odds ratios (OR) and 95% confidence intervals (CI) for polypharmacy in relation to job demands and control.

Job demands/control	Model 1 ^a^			Model 2 ^b^		
	
	OR	95% CI	P-value	OR	95% CI	P-value
Job control (continuous)	0.79	0.65–0.98	0.033	0.82	0.64–1.04	0.100
Job demands (continuous)	1.00	0.83–1.21	0.991	1.06	0.87–1.28	0.576
Job control						
1^st^ quartile (lowest)	1			1		
2^nd^ quartile	0.73	0.57–0.94	0.016	0.73	0.56–0.94	0.053
3^rd^ quartile	0.68	0.52–0.90	0.006	0.69	0.52–0.93	0.013
4^th^ quartile (highest)	0.77	0.58–1.01	0.060	0.81	0.60–1.09	0.170
Job demands						
1^st^ quartile (lowest)	1			1		
2^nd^ quartile	1.09	0.82–1.45	0.538	1.15	0.86–1.53	0.354
3^rd^ quartile	1.00	0.78–1.28	0.984	1.05	0.82–1.35	0.702
4^th^ quartile (highest)	0.94	0.71–1.23	0.646	0.99	0.74–1.31	0.920
Job control						
Low (1^st^ quartile)	1			1		
High (2^nd^ – 4^th^ quartile)	0.73	0.59–0.89	0.002	0.74	0.59–0.92	0.007
Job demands						
Low (1^st^ - 2^nd^ quartile)	1			1		
High (3^rd^ – 4^th^ quartile)	0.95	0.78–1.15	0.587	0.98	0.80–1.19	0.835

a Adjusted for age, sex, and follow-up time.

b Adjusted for age, sex, education, follow-up time, occupational class and number of chronic diseases.

**Table 4:** Odds ratios (OR) and 95% confidence intervals (CI) for polypharmacy in relation to job strain

Job strain	Model 1 ^a^			Model 2 ^b^		
	
	OR	95% CI	P-value	OR	95% CI	P-value
Low strain ^c^	1			1		
Active job ^d^	0.86	0.68–1.69	0.223	0.89	0.70–1.14	0.349
Passive job ^e^	1.19	0.90–1.58	0.228	1.19	0.89–1.60	0.242
High strain ^f^	1.40	1.05–1.88	0.024	1.40	1.04–1.89	0.029

a Adjusted for age, sex and follow-up time.

b Adjusted for age, sex, education, follow-up time, occupational class and number of chronic diseases.

c High control and low demands.

d High control and high demands.

e Low control and low demands.

f Low control and high demands.

In the sensitivity analysis, we found similar results among individuals ≥26 years (high-strain jobs: OR 1.41, P=0.025), in stratified analysis by age (age <55, high-strain jobs: OR 1.42, P=0.081 and age ≥54, high-strain jobs: OR 1.40, P=0.164), and in stratified analysis by occupation (among blue-collar workers, high-strain jobs: OR 1.30, P=0.239 and among white-collar workers, high-strain jobs: OR 1.52, P=0.062). When using active jobs as the reference group, results remained similar in fully adjusted models (high-strain jobs: OR 1.57, 95% CI 1.15–2.16).

**Figure 1:**
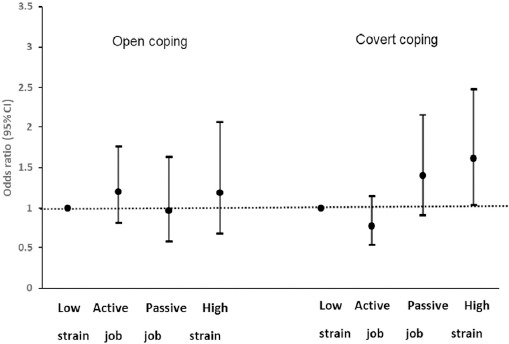
Odds ratios (OR) and 95% confidence intervals (CI) for the relationship of job strain and polypharmacy by coping strategy adjusted for age, sex, education, follow-up time, occupational class, self-rated health, and number of chronic diseases.

We detected a statistically significant interaction between coping strategy and high-strain jobs. After stratifying by coping strategy, the effects of high job strain on developing polypharmacy remained in those with covert coping strategies (OR 1.61, 95% CI 1.04–2.48) (figure 1). However, no association was observed among those with open coping strategies. Similar results were observed after excluding individuals younger than 26 years, among those with covert coping strategies (high-strain jobs: OR 1.63, P=0.026) and among those with open strategies (high-strain jobs: OR 1.19, P=0.52). Among white-collar workers, passive jobs were associated with an increased risk of polypharmacy in those with covert coping strategies (OR 1.95, P=0.026). When using active jobs as the reference group, high-strain jobs (OR 2.05, 95% CI 1.32–3.21) and passive jobs (OR 1.79, 95% CI 1.15–2.79) were significantly associated with polypharmacy in those with covert coping strategies. No association was found for those with open coping strategies.

These results did not remain significant in other sensitivity analyses, when participants taking any drugs at baseline were excluded, and when the occurrence of multiple/repeated polypharmacy was used as the outcome (supplementary tables S1 and S2). In sensitivity analysis 2, active jobs were associated with an increased risk of polypharmacy compared to low-strain jobs (OR 1.99, 95% CI 1.07–3.72) in those with open coping strategies.

## Discussion

Our study found that when compared with workers in low-strain jobs, high-strain jobs were associated with an increased risk of polypharmacy. After stratifying by coping strategies, this association remained only in those with covert coping strategies.

Previous studies have found job strain to be associated with a range of individual chronic diseases (12, 13, 15, 18) and shorter health expectancy (36). However, to our knowledge, no previous studies have looked at the association between job strain and polypharmacy. Polypharmacy indicates clinically significant medical conditions and symptoms requiring pharmacological intervention and provides a marker of multimorbidity and disease severity. However, polypharmacy may also indicate potential overuse of medications, including inappropriate or problematic medications and combinations. In such situations, the harms of medications may outweigh the intended benefits (1).

Our study found that high control at work was associated with a lower risk of polypharmacy, while job demands alone did not have a significant impact. This is in line with previous studies of other health outcomes including cardiovascular disease (13), mental health (37) and dementia (38). Our finding highlights the importance of control at work despite level of demands, suggesting that workers who are allowed to make decisions or utilize skills to manage work demands have lower levels of stress and thus avoid multiple medication use.

In our study, we found that compared with low-strain jobs, those with high-strain jobs were at greater risk for developing polypharmacy. These associations remained after adjusting for a range of potential confounders, including occupational and health conditions, suggesting a direct association between job strain and polypharmacy. Low-strain jobs are theoretically ideal jobs, being the most relaxed, and were used as the comparator in our main analysis. Conversely, high-strain jobs, where low control is combined with high demands, represent stressful work situations, thus placing workers at risk of adverse health events. We used active jobs as a reference group in our secondary analysis. Active jobs are highly demanding jobs that allow the employee to decide when they do their work. As a result of the high level of decision latitude, employees do not experience their job as stressful, despite it being very psychologically demanding (11). Interestingly, both high-strain and passive jobs were associated with an increased risk for polypharmacy compared to active jobs in those with covert coping strategies. Passive jobs, where low control is combined with low demands, can be demotivational to workers as their skills and self-efficacy are underutilized and diminished (39). Under stimulation at work may also be recognized as a source of stress, similar to overstimulation (40). Thus, passive jobs can also lead to chronic stress. Passive jobs have previously been demonstrated to be associated with disability, multimorbidity and other outcomes (41, 42).

There are potential physiological mechanisms that may explain the observed association between psychosocial job strain and polypharmacy. Firstly, chronic stress may lead to neuroendocrine dysregulation of the hypothalamic-pituitary-adrenal axis, which in turn induces changes in the immune and inflammation systems and consequently the development of several chronic conditions and polypharmacy (12, 43, 44). These processes have been conceptualized as allostatic load (45). Work stress has also been linked to a range of disease risk factors including increased adiposity, systemic inflammation, and altered metabolic profile (46). Secondly, stress has been identified as a significant feature of several mental illnesses, including depression (12, 44), which may explain increased use of medications, especially psychotropics. Finally, stress may also lead to unhealthy lifestyle behaviors, such as smoking, heavy alcohol consumption, and physical inactivity (47, 48), which may also be implicated in the development of cardiometabolic diseases and polypharmacy. However, we found no significant interaction between lifestyle factors and job strain in our study.

Coping strategies appeared to moderate the association between psychosocial job strain and polypharmacy. In our study, more open styles of coping ameliorated the association between job strain and polypharmacy, with the association remaining only in those with more covert coping strategies. Previous studies have demonstrated that covert coping with unfair treatment at work is associated with an increased risk of cardiovascular disease (23, 24). Work-related stress in the presence of covert coping may also increase risk for oesophageal and gastric cancers (49). This highlights the important interaction between a stressful work environment and an individual’s response to it. It is postulated that poor coping strategies can result in psychophysiological tension and the development of illness (24), which in turn may lead to polypharmacy. In subgroup analysis by occupational class, passive jobs were associated with an increased risk of polypharmacy among white-collar workers with covert coping strategies. This may reflect poor health behaviors and coping such workers (50).

The impact of high-strain jobs on polypharmacy disappeared when we took into account any drug use at baseline and repeated occurrences of polypharmacy at follow-up. This may suggest that the influence of job strain on polypharmacy is greatest in those with underlying chronic health problems but may not be long lasting. Interestingly, active jobs were associated with repeated occurrences of polypharmacy among workers with open coping strategies, suggesting potential stress from active jobs. More studies are warranted to clarify the underlying mechanisms.

The most commonly used medications contributing to polypharmacy reflect the treatment of medical conditions previously found to be associated with job strain including cardiovascular disease (beta blockers, lipid modifying agents, antithrombotics, ACE inhibitors), mental disorders (antidepressants, hypnotics and sedatives), gastroesophageal disease (drugs for peptic ulcer and gastro-oesophageal disease), and musculoskeletal and pain disorders (opioids and other analgesics). However, previous studies investigating the association between job strain and specific medication use have been inconsistent. A previous study demonstrated that psychological work demands were associated with the purchase of antihypertensive medication in women (51). Exposure to low job control and high job demands was associated with subsequent psychotropic medication use, including antidepressants and anxiolytics, in a previous study (52). Another study found that men with high job strain had an increased risk of future antidepressant medication use (53). A cross-sectional study found an association between high job strain and self-reported benzodiazepine use (54). Conversely, other previous studies have found no significant association between job strain and psychotropics, including antidepressants (55).

Our study findings highlight the need for organizations and workplaces to optimize psychosocial work conditions in order to potentially reduce future risk of ill health and subsequent polypharmacy. Policies to empower workers to adopt better and more open coping strategies to mitigate this risk should be encouraged. Future studies should look at other types of working conditions, as well as the interplay between different medication classes. Strategies to manage psychosocial job strain at work should be further explored. As working conditions continue to evolve, and multimorbidity and complex drug regimens become more common with an aging population, such strategies will be of great importance to ensure a healthy and sustainable workforce.

This study has strengths and limitations. Strengths lie in the large, representative sample of Swedish workers. The prospective study design allowed a temporal association between psychosocial job strain and incident polypharmacy to be explored; however, causality cannot be concluded. Medication outcomes and medical comorbidity data were ascertained from national registers that were complete, thus avoiding any potential attrition or recall bias. However, data on psychosocial work environment and covariates were self-reported, which may lead to misclassification and response bias. Non-response varied from 35–39%; as occupational status is unknown among non-responders, it is difficult to determine whether respondents represent the overall Swedish working population. However, it was previously shown that, in general, those who were women, younger, married, university educated and born in Sweden were more likely to respond to the SLOSH questionnaires (28). Thus, this may impact the generalizability of our findings. We did not consider non-prescription medications such as those obtained over-the-counter, thus we may have underestimated incidence of polypharmacy. The measure of coping used in this study was relatively crude and other factors could explain the observed associations, such as personality traits or other personal characteristics underlying coping patterns (24). Additionally, this measure relates mainly to unfair treatment or conflicts at work and may not be optimal for other types of work stress. In addition, the large internal missing rate of the coping questions (13%) is a potential weakness. Although we adjusted for a range of important covariates, we cannot exclude the possibility of bias due to unmeasured confounding.

### Concluding remarks

Workers of high-strain jobs may be at an increased risk of polypharmacy. Open coping strategies may reduce the negative effects of psychosocial job strain on risk of polypharmacy.

## Supplementary material

Supplementary material
