# Supplementary material for: Psychosocial job strain and polypharmacy: a national cohort study
Source: Scand J Work Environ Health. 2020 Oct 30;46(6):589–98. doi: 10.5271/sjweh.3914 (PMC7737808; doi:10.5271/sjweh.3914)
Supplement: Supplementary material [file SJWEH-46-589-S001.pdf]

Psychosocial job strain and polypharmacy: a national cohort study [1]

by Edwin CK Tan, PhD,[2] Kuan-Yu Pan, PhD, Linda L Magnusson Hanson, PhD, Johan Fastbom, PhD, Hugo Westerlund, PhD, Hui-Xin Wang, PhD

1. *Supplementary Material*
2. *Correspondence to: Edwin CK Tan, Stress Research Institute, Department of Psychology, Stockholm University, Stockholm, Sweden.*
   *[E-mail: edwin.tan@sydney.*edu.au]

Appendix: *Other covariates*

General self-rated health was measured by the one-item question: 'How would you rate your general state of health?'. Respondents answered on a scale from 1 = 'very good' to 5 = 'very poor'. Self-rated health was categorized into good ('very good' or 'good'), neither good nor bad, and bad ('quite poor' or 'very poor').

Sleep disturbances during the past three months were assessed with four questions from the Karolinska Sleep Questionnaire (difficulty falling asleep, repeated awakenings, early awakening, and disturbed sleep); whereas awakening problems were measured with 3 questions: difficulty awakening, not well-rested, and exhausted at awakening, following the Karolinska Sleep Questionnaire in SLOSH [1]. The occurrence and frequency of problems were quantified on a scale from 1 = 'never' to 6 = 'always', five times a week or more. Sleep disturbances were defined as the presence of at least one of the symptoms three to four times a week or more, in line with some of the diagnostic criteria for insomnia.

Depressive symptoms were assessed in SLOSH with a brief subscale (6-item) from the Hopkins Symptom Checklist 90 (SCL-90), which assesses intensity of being troubled by: feeling blue; feeling no interest in things; feeling lethargy or low in energy; worrying too much about things; blaming yourself for things; and feeling everything is an effort over the past week. The intensity was quantified on a five-category scale from 1 = 'not at all' to 5 = 'extremely'. These items represent core symptoms and the scale has been shown to have good psychometric properties [1]. A dichotomous variable for major depressive symptoms was derived using a cut-off of 17.

Lifestyle factors included smoking, alcohol intake and physical activity. In SLOSH, self-reported smoking was categorized as 'yes' if the response was currently smoking (every day or occasionally) and 'no' for those who did not smoke. Alcohol consumption was categorized as 'heavy' (more than 2 glasses of alcohol more often than 4 days a week), 'moderate' (1-2 glasses of alcohol less often than 4 days a week), and 'no' (no drinking at all or less than once a month). Physical activity was measured by asking: "How much exercise do you get?" and was dichotomised as 'regular physical activity' versus 'physical inactivity' (no or very little exercise, only occasional walks).

References

Hanson LLM, Akerstedt T, Naswall K, Leineweber C, Theorell T, Westerlund H. Cross-Lagged Relationships Between Workplace Demands, Control, Support, and Sleep Problems. Sleep 2011; 34: 1403-U147.

Supplementary Table S1. Odds ratios (ORs) and 95% confidence intervals (CIs) for polypharmacy in relation to job strain among those who did not take any drugs at baseline (sensitivity analysis 1)

Job strain	Model 1[a]			Model 2[b]		
	OR	95% CI	*p*	OR	95% CI	*p*
Whole population						
Low strain	1			1		
Active job	0.68	0.47-0.96	0.030	0.71	0.49-1.01	0.059
Passive Job	1.24	0.83-1.84	0.290	1.23	0.82-1.86	0.321
High strain	1.34	0.89-2.03	0.163	1.31	0.86-2.01	0.213
Covert coping						
Low strain	1			1		
Active job	0.62	0.36-1.06	0.081	0.64	0.36-1.11	0.116
Passive Job	1.55	0.89-2.71	0.120	1.68	0.93-3.01	0.086
High strain	1.48	0.84-2.60	0.173	1.60	0.89-2.89	0.120
Open coping						
Low strain	1			1		
Active job	0.86	0.48-1.56	0.628	0.94	0.51-1.73	0.845
Passive Job	1.33	0.66-2.69	0.421	1.20	0.59-2.46	0.615
High strain	1.35	0.61-3.00	0.455	1.15	0.52-2.57	0.732

[a] Adjusted for age, sex, and follow-up time.
[b] Adjusted for age, sex, education, follow-up time, occupational class, and number of chronic diseases.

Active job: high control and high demands; low strain: high control and low demands; passive job: low control and low demands; high strain: low control and high demands

Supplementary Table S2. Odds ratios (ORs) and 95% confidence intervals (CIs) for multiple/repeated polypharmacy occurrences in relation to job strain (sensitivity analysis 2)

Job strain	Model 1[a]			Model 2[b]		
	OR	95% CI	*p*	OR	95% CI	*p*
Whole population						
Low strain	1			1		
Active job	1.03	0.72-1.48	0.873	1.06	0.73-1.54	0.746
Passive Job	1.30	0.84-2.02	0.235	1.33	0.84-2.10	0.224
High strain	1.28	0.80-2.05	0.57	1.36	0.84-2.20	0.217
Covert coping						
Low strain	1			1		
Active job	0.81	046-1.42	0.453	0.81	046-1.45	0.479
Passive Job	1.46	0.79-2.71	0.226	1.52	0.80-2.90	0.198
High strain	1.39	0.73-2.63	0.312	1.48	0.77-2.85	0.245
Open coping						
Low strain	1			1		
Active job	1.82	1.00-3.32	0.048	1.99	1.07-3.72	0.030
Passive Job	1.12	0.47-2.63	0.701	1.19	0.49-2.87	0.701
High strain	1.21	0.46-3.20	0.708	1.21	0.45-3.29	0.708

[a] Adjusted for age, sex, and follow-up time.
[b] Adjusted for age, sex, education, follow-up time, occupational class, and number of chronic diseases.

Active job: high control and high demands; low strain: high control and low demands; passive job: low control and low demands; high strain: low control and high demands
